# Drug adherence for antihypertensive medications and its determinants among adult hypertensive patients attending in chronic clinics of referral hospitals in Northwest Ethiopia

**DOI:** 10.1186/s40360-017-0134-9

**Published:** 2017-04-05

**Authors:** Habtamu Sewunet Mekonnen, Mignote Hailu Gebrie, Kokeb Haile Eyasu, Abebaw Addis Gelagay

**Affiliations:** 1 Department of Medical Nursing, College of Medicine and Health Science, University of Gondar, Gondar, Ethiopia; 2 Institute of Public Health, College of Medicine and Health Science, University of Gondar, Gondar, Ethiopia

**Keywords:** Antihypertensive medication, Drug adherence, Ethiopia, Hypertensive patients

## Abstract

**Background:**

Adherence to prescribed medication is an imperative issue which can be directly linked with the management of chronic diseases like hypertension; failure to adhere can affect the effectiveness of medication as well as the efficiency of the health care system. There is scarcity of information regarding the level of drug adherence for antihypertensive medications and its determinants in Ethiopia, particularly in the study area. Therefore, the aim of this study was to assess adherence level and its determinants for antihypertensive medications among adult hypertensive patients attending the chronic illness clinics of the referral hospitals in northwest Ethiopia.

**Methods:**

Institution based cross sectional study was conducted from March to April, 2016. The systematic random sampling technique was used to select 409 study participants from three referral hospitals. The questionnaire was prepared using the World Health Organization (WHO) conceptual model and by reviewing international literature. The data were collected using an interviewer administered questionnaire. The data were entered in to Epi - Info version 7 and then transferred to the statistical package for social science (SPSS) version 20 for data cleaning and analysis.

Bivariate analysis was first done to see the association between each independent variables and dependent variable. Variables with a P-value of less than 0.2 in the bivariate analysis were entered in to the multivariate logistic regression model for final analysis. Multivariate analysis was done using Backward logistic regression method.

*P*-value less than 0.05 was considered to determine the statistical significance of the association and odds ratio with a 95% confidence interval was used to determine the presence, strength, and direction of association between covariates (explanatory variables) and the outcome variable. The Morisky medication adherence scale was used to assess the adherence status using > = 6 as adherent or < 6 as non adherent score.

**Results:**

Four hundred and nine (409) study participants were interviewed with a response rate of 100%. The mean age of the respondents was 54.5 years with (Standard Deviation (SD) ± 13.58). The overall rate of good adherence was 67.2% (95% CI = 62.8, 71.6). Participants who had a favourable attitude towards antihypertensive medications (Adjusted odds ratio (AOR) = 9.88, 95% confidence interval (CI): 5.34, 18.27), having good patient- provider relationship (AOR = 4.25, 95% CI: 2.32, 7.86), having one (AOR = 4.36, 95% CI: 1.34, 14.12) or no (AOR = 3.38, 95% CI:1.01,11.31) co-morbidities, a long duration of treatment (AOR = 1.89, 95%CI: 1.07, 3.35), and a low medical cost (AOR = 2.06, 95% CI: 1.13, 3.76) had associations with good drug adherence for antihypertensive medication/s.

**Conclusions:**

The prevalence of good drug adherence for anti-hypertensive medications in this study was high. Prevention of co- morbidities, making medical services accessible, and maintaining good client-provider interaction are of paramount importance for good drug adherence.

## Background

Hypertension, high or increased blood pressure (the systolic and diastolic level ≥ 140/90 mmHg), is a worldwide public health problem. It contributes to the burden of cardiovascular diseases, stroke, and renal failure leading to early mortality and disability [1].

Global prevalence of adult hypertensive patients was about 22% in 2014 [2]. It accounted an estimated more than 40% in African adult population [3]. In Sub Saharan Africa, it varied from 6% to 48%, that is 74.7 million hypertensive individuals which will be 68% (125.5 million) by 2025 [4, 5]. In Ethiopia, it has a widely varied rate ranging from 0.8% to 31.5% [6]. The rate was 28.3% and 25.1% for Gondar town [7] and Bahir Dar [8] respectively.

Uncontrolled hypertension is still a big medical and psychosocial problem in developed as well as developing countries. Even if the risk factors, prevention, and controlling mechanisms are well familiar, the negative outcomes resulting from the disease will possibly continue for many years. This makes the disease the biggest and most terrible social and health related challenges [9].

Hypertension (HTN) is a modifiable cardiovascular risk factor for which medications effective to regulate the raised blood pressure as well as to hamper the complications are available. But, the maximal beneficial effect of an appropriate treatment plan can be achieved only if patients strictly adhere to the recommendations. The study done by a high performance liquid chromatography –tandem mass spectrometry urine analysis shows the highest prevalence of partial and total non -adherence among follow up patients with inadequate blood pressure control, and there was a linear relationship between blood pressure (BP) level and prescribed antihypertensive medications [10].

Poor adherence to antihypertensive medications is an obstacle in the management of hypertension resulting in high rate of hospitalization and death [11, 12]. It undermines the efforts of health facilities, health professionals, and policy makers for the improvement and modification of the health of the people. Poor adherence will be the source of psychological and medical complications and has an impact on patients’ quality of life, wasting health care resources and reducing individual's believe towards the health care system [13]. The study at tertiary hospital in Nigeria showed that patients with poor antihypertensive adherence had higher Sokolow-Lyon ECG (electro cardiograph) score, as well as longer P wave duration on ECG and higher rate of ECG LVH(left ventricular hypertrophy) compared to patients with good drug adherence [14].

Generally, good adherence to medications is an important achievement in disease management, and it is crucial to decrease complications like cardiovascular related morbidity and mortality [15, 16]. However, there is a scarcity of information regarding the level of drug adherence for antihypertensive medications and its determinants in Ethiopia, particularly in the study area. Therefore, this study aimed to assess adherence status and associated factors for anti-hypertensive medications among adult hypertensive patients attending the chronic illness clinics of referral hospitals in northwest Ethiopia.

## Methods

### Study design and period

Institution based cross sectional quantitative study was conducted in northwest Ethiopia referral hospitals from March to April, 2016.

### Study area

The study was conducted in referral hospitals in northwest Ethiopia, namely Debre Markos Referral Hospital at Debre Markos town, Felege Hiwot Referral Hospital at Bahir Dar city and Gondar university referral Hospital at Gondar city. Debre Markos and Gondar, which are 300 Km and 748 Km, respectively from Addis Ababa, the capital city of Ethiopia, to the northwest are zonal cities of the Amhara National Regional state. Bahir Dar, the capital of the region, is 565 km from Addis.

### Source and study population

All adult hypertensive patients who were attended the chronic illness clinics of the referral hospitals in northwest Ethiopia were the source and the study population.

### Inclusion criteria

Hypertensive patients aged ≥18 years who have been taking antihypertensive medications at least for a month were included in the study.

### Exclusion criteria

Individuals who were not capable of hearing and speaking and had known mental disorders or serious illness were excluded from the study.

### Sample size and sampling procedure

The sample size was computed by using the single population proportion formula (n = [(Zα/2)^2^ × P (1-P)]/D^2)^ with the assumption of a 95% level of confidence, 5% margin of error, taking prevalence of adherence to antihypertensive medications as 59.5% in Adama study [17] and adding a 10% non response rate. Based on these assumptions, the final sample size was 409. The total samples were proportionally allocated to the three referral hospitals. Study participants were selected using systematic random sampling. In each hospital, every 6 participants were interviewed based on their order of arrival.

### Data collection tool and procedure

Adherence status was assessed using the Morisky medication adherence scale −8 which is a self reporting method to determine adherence. It contains eight questions with seven closed dichotomous (yes/no) answers and one liker scale question. Each item measures specific adherence behaviour. The degree of adherence was determined according to the score resulting from the sum of all the correct answers [18].

Questions regarding the explanatory variables were prepared using the WHO conceptual model and by reviewing international literature. The questionnaire was first developed in English and translated to Amharic, the local language, and then retranslated to English. A pre-test was administered on 21 patients at Debre Tabor Regional Hospital, northwest Ethiopia. Some amendment was made on the tool after the pre-test. In each Referral Hospital, one MSc and two trained BSc graduate nurses who were working out of the chronic illness clinics were employed for supervision and data collection, respectively. The data collection technique was a face to face interview by using a structured questionnaire. Important data like, BP were also taken by reviewing respondents’ medical records or documents. After obtaining a written informed consent, the required data were collected on isolated place. To avoid the chance of recycling of data, a special mark was placed on each medical chart.

### Data quality assurance

Data was controlled by using structured questionnaire which was translated to the local language, Amharic, and then translated back to English so as to see the consistency. The tool was pre-tested before use. Two days training was given for data collectors and supervisors on the tool and the procedure. Intensive supervision was carried out and data were checked for completeness and accuracy by each supervisor.

### Operational definition

**Adherent**: respondents who scored ≥ 6 points of the Morisky medication adherence scale - 8 [19].

**Control Hypertension**: maintaining the average BP reading less than 140/90 mmHg at the time of data collection irrespective of measurements at other time.

**Knowledgeable**: when respondents scored points at mean and above (> = 6) for the knowledge question prepared on hypertension (HTN).

**Favourable attitude**: respondents who scored points at mean and above (> = 3) for the attitude questions prepared on HTN and its treatment were referred to be favourable.

**Good patient**-**provider relationship**: participants who scored points at mean and above (> = 6) for questions prepared on patient provider relationship in the treatment and care of HTN patients.

**Good life style**: participants who scored points at mean and above (> = 4) for questions prepared on life style modification in the treatment and care of HTN patients otherwise not.

**Co**-**morbidity**: the presence of any of the chronic disease along with hypertension.

### Data processing and analysis

The data were checked for completeness and coded manually. After coding, it was fed to Epi info version 7. The Statistical package for social sciences (SPSS) version 20 was used for data analysis. Descriptive statistic including frequencies, means and standard deviations were calculated. Each variable was first analyzed by using bivariate logistic regression (bivariate analysis) and independent variables having P-value of less than 0.2 were entered in to multivariate logistic regression model for final analysis. Multivariate analysis was done using Backward logistic regression method. In multivariate analysis, variables with a *P*- value of ≤ 0.05 were considered as statistically significant. Adjusted odds ratio (AOR) was computed to see the strength and direction of the association between dependent and independent variables.

## Results

### Socio-demographic characteristics of the study participants

In this study, a total of 409 study participants were interviewed with a response rate of 100%. The mean age of respondents was 54.5 years with a standard deviation (SD) of ±13.58. Most of the respondents, 331 (80.9%), were Orthodox Christian by religion. Amhara ethnic groups accounted for 333 (81.4%) of the total study participants. More than three-fourth (78.2%) of the respondents were urban dwellers. Two hundred sixty one (63.8%) reported as married, and 105 (25.7%) were government employees. Two hundred twenty-two (54.3%) of the respondents took less than half an hour (single trip) to reach to the hospital (Table 1).

**Table 1:** Socio-demographic Characteristic of hypertensive patients attending care in referral Hospitals in Northwest Ethiopia, 2016 (*n* = 409)

Variable	Frequency	Percent (%)
Sex		
Male	236	57.7
Female	173	42.3
Age		
18–38	57	13.9
39–59	193	47.2
> = 60	159	38.9
Marital status		
Single	39	9.5
Married	261	63.8
Divorced	47	11.5
Widowed	62	15.2
Religion		
Orthodox	331	80.9
Muslim	62	15.2
Protestant	16	3.9
Ethnicity		
Amhara	333	81.4
Oromo	16	3.9
Tigrie	33	8.1
Kimant	27	6.6
Residence		
Urban	320	78.2
Rural	89	21.8
Educational status		
Unable to read and write	150	36.7
Able to read and write	57	13.5
Grade 1 – 8	66	16.1
Grade 9 – 12	69	16.9
Above secondary school	67	16.4
Occupational status		
Government	105	25.7
Merchant	58	14.2
Farmer	61	14.9
Housewife	104	25.4
Non employed	21	5.1
Retired	50	12.2
Others^a^	10	2.4
Distance from the hospital(single trip)		
< = 0.5 h	222	54.3
> 0.5 h	187	45.7
Wealth status		
Poor	130	31.8
Medium	156	38.1
Rich	123	30.1

^a^ Others in occupation: include daily labourer, garden**,** priest, NGO and private work

### Setting, characteristics of hypertension and antihypertensive treatments of the respondents

In this study, 246 (60.1%) of the participants had uncontrolled blood pressure. Regarding the treatment, more than half of the participants, 234 (57.2%), were on treatment for greater than 3 years, and 231(56.5%) were on mono-therapy. About two- third of the participants, 267(65.3%), had no co-morbidity (Table 2).

**Table 2:** Setting, clinical and treatment related characteristic of respondents, Northwest Ethiopia referral hospitals, 2016 (*n* = 409)

Variables	Frequency	Percent
Setting/hospitals/		
Gondar university hospital	154	37.7
Debre Markos hospital	67	16.3
Felege Hiwot hospital	188	46.0
Blood Pressure status (systolic/diastolic)		
Uncontrolled	246	60.1
Controlled	163	39.9
Duration since diagnose of hypertension		
< 4 years	285	69.7
> = 4	124	30.3
Duration since starting treatment		
< 3 years	175	42.8
> = 3 years	234	57.2
Number of anti hypertensive drugs:		
Mono therapy	231	56.5
Two drugs	148	36.2
Three drugs and above	30	7.3
Number of tabs per day:		
One	206	50.4
Two	121	29.6
Three and more	82	20
Dosage frequency:		
Once daily	225	55
Two times/day	164	49.9
Three times and above	20	4.2
Medical cost per month(birr) for hypertension		
< =50	174	42.5
> 50	235	57.5
Co- morbidity		
Yes	142	34.7
No	267	65.3
Number of co morbidity		
None	267	65.3
One	120	29.3
Two	22	5.4

### Knowledge and attitude of respondents towards hypertension and its treatment and patient-provider relationship

About two-third of the respondents, 258 (63.1%), were knowledgeable and 287 (70.2%) had favourable attitude towards hypertension (HTN) and its treatment. Two hundred sixty eight (65.5. %) participants had good patient-provider relationship.

### Respondents’ level of adherence to antihypertensive medications

**Fig. 1:**
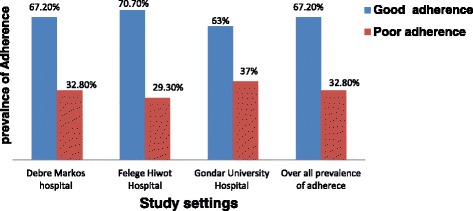
Respondents level of adherence to antihypertensive medications among hypertensive patients attending care in three referral Hospitals in Northwest Ethiopia

In this study, the overall prevalence of good drug adherence to antihypertensive medications was 67.2% (95% confidence interval (CI) = 62.8, 71.6). Specifically, the prevalence of drug adherence among respondents in the three hospitals is shown in fig. 1.

### Factors associated with adherence to antihypertensive medications

The multivariate analysis revealed that having co-morbidity, duration of treatment, medical cost, attitude, and patient-provider relationship were significantly associated with adherence to antihypertensive treatment. Accordingly, study participants who had no co- morbidity were four times (AOR = 4.36, 95%CI = 1.34, 14.11) more likely to adhere to antihypertensive medications than their counterparts.

Respondents who were on antihypertensive medications for three and more years were two times (AOR = 1.89, 95% CI =1.10, 3.35) more likely to adhere to antihypertensive medications compared to those who were on antihypertensive medications for less than three years. Moreover, the odds of adherence to antihypertensive medications was 2 times (AOR = 2.06, 95% CI = 1.13, 3.76) higher in respondents who had got the medication/s free of charge or with low cost as compared to those who had got the medication/s with high cost.

Participants who had favorable attitude about antihypertensive treatment were ten times (AOR = 9.88, 95% CI = 5.34, 18.27) more likely to adhere to antihypertensive medications than those who had unfavorable attitude. Besides, the odds of good adherence to antihypertensive medication was 4 times (AOR = 4.27, 95% CI = 2.32, 7.86) higher among respondents with good patient –provider relationship compared to poor patient- provider relationship (Table 3).

**Table 3:** Bivariate and multivariate analysis for level of adherence to antihypertensive drugs among hypertensive patients attending care in referral Hospitals in Northwest Ethiopia, 2016 (*n* = 409)

Variables	Adherence status		COR(95% CI)	AOR(95% CI)
	Adherent	Non adherent		
Age				
18–38	37	20	1	1
39–59	125	68	0.99(0.54,1.84)	0.72(0.28,1.82)
> = 60	113	46	1.33(0.70,2.53)	0.72(2.33,2.21)
Marital status				
Single	26	13	1.44(0.63,3.33)	0.78(0.23,2.62)
Married	188	73	1.86(1.05,3.30)*	1.25(0.52, 3.04)
divorced	25	22	0.82(0.38,1.76)	0.76(0.25,2.38)
Widowed	36	26	1	1
Residence				
Urban	213	107	1	1
Rural	62	27	1.15(0.69,1.92)	0.69(0.35,1.39)
Educational status				
Unable to read and write	108	42	1	1
Able to read and write	39	18	0.84(0.43,1.63)	1.27(0.45,3.58)
Grade 1–8	34	32	0.41(0.23,0.75)*	0.82(0.31,2.18)
Grade 9-12	45	24	0.73(0.40,1.34)	1.52(0.52,4.46)
Above secondary school	49	18	1.06(0.55, 2.02)	0.75(0.16,3.43)
Occupation				
Government	77	28	1	1
Merchant	35	23	0.55(0.28,1.09)	0.99(0.39,2.55)
Farmer	38	23	0.60(0.31,1.18)	0.33(0.09,1.17)
House wife	70	34	0.75(0.41,1.36)	0.87(0.37,2.04)
Others	55	26	0.77(0.41,1.45)	0.5(0.21,1.18)
Distance from the hospital(single trip)				
< =1/2 h	150	72	1.03(0.68,1.56)	0.89(0.48,1.64)
> ½ hour	125	62	1	1
Wealth status				
Poor	81	49	1	1
Medium	110	46	1.45(0.88,2.37)	0.92(0.44,1.92)
Rich	84	39	1.30(0.78, 2.19)	1.05(0.36,3.08)
Setting/Hospitals/				
Gondar University	97	57	1	1
Debre Markos	45	22	1.20(0.66,2.20)	1.28 (0.57, 2.81)
Felege Hiwot	133	55	1.42(0.90,2.24)	1.31 (0.71,2.41)
No of co morbidities				
None	180	87	2.07(0.86,4.96)	4.36(1.34,14.12)**
One	84	36	2.33(0.93,5.87)	3.38(1.01,11.31)**
Two or more	11	11	1	1
B/P				
Not controlled	163	83	1	1
Controlled	112	51	1.12(0.73,1.71)	1.07(0.55,2.06)
Duration since starting treatment				
< 3 years	99	76	1	1
> = 3 years	176	58	2.33(1.53,3.55)*	1.89(1.1,3.35)**
Antihypertensive drugs				
One	159	72	1	1
Two	95	53	0.81(0.53,1.26)	1.97(0.76,5.09)
Three and above	21	9	1.06(0.46,2.42)	3.03(0.64,14.29)
Number of tabs per day:				
One	150	56	1.55(0.90,2.66)	2.09(0.97,4.53)
Two	73	48	0.88(0.49,1.57)	1.1(0.48,2.37)
Three and more	52	30	1	1
Dosage Frequency				
Once daily	162	63	1.62(1.07,2.45)*	1.52(0.76,3.02)
Twice or more	113	71	1	1
Medical cost per month (birr) for hypertension				
< 50 birr	130	44	1.83(1.12,2.82)*	2.06(1.13,3.76)**
> = 50 birr	145	90	1	1
Knowledge				
Knowledgeable	207	51	4.95(3.18,7.72)*	1.45(0.78,2.7)
Not Knowledgeable	68	83	1	1
Attitude				
Favourable	245	42	17.89(10.57,30.28)*	9.88(5.34,18.27)**
Unfavourable	30	92	1	1
Pt-provider relationship				
Good	224	44	8.98(5.61,14.40)*	4.27(2.32,7.86)**
Poor	51	90	1	1

* Variables those were significant during bivariate logistic analysis at *P* value 0.05

** Variables that was found to have significant association during multivariate analysis at *p* value < 0.05

## Discussion

In many developing countries, maintaining good adherence to antihypertensive medications remained the most important challenge. Adherence to antihypertensive medications contributes for controlled blood pressure and the prevention of complications.

About two-third, 67.2% (95% CI = 62.8, 71.9), of the study participants had good adherence to antihypertensive medications. This finding is in-line with other studies done at Gondar University Hospital (64.6%) [20] and in the new territories region of Hong Kong (65.1%) [21].

However, the finding is higher than what was reported in the study done in Adama Referral Hospital (59.5%) [17], Dar es Salaam (56%) [22], Eastern Nigeria (52.5%) [23], Southwest Nigeria (51%) [24], and Hong Kong (55.1%) [25]. This might be due to differences in socio-demographic characteristics, small sample size in Dar es Salaam and time difference. The other possible justification could be enrolment criteria and study area difference. In Hong Kong study it was in clinics where as this study is Hospital based. In addition, data collection technique in Hong Kong study was self administered but in this study it was interviewer based.

The finding is lower than the studies conducted in Maritime Canada (77.0% [26], Pakistan(77.0%) [27], Singapore (75.2%) [28] and in Sunderland (79%) [29]. This difference could be due to difference in socio-demographic characteristics and health care facilities among this study and the comparison studies. According to WHO, a health service is not accessible if it takes one hour and above of round trip [30]; in this study, for 45.5% of the participants it takes more than one hour to get a service which could lead to missing of appointments. Moreover, in Sunderland study participants who were taking only anti hypertensive medication for greater than 6 months and merely taking antihypertensive drugs were included.

While compared to associations between outcome and explanatory variables, significant association was observed between adherence status and the number of co- morbidities was. This finding is supported by studies conducted in Adama Hospital [17], Gondar University Hospital [20], Bangladesh [31] and Hong Kong [25]. The adherence to antihypertensive medication was increased when there was no co- morbidity. The possible reason could be if there is co –morbidity, they would have pill burden and if drugs are taken orally, they may cause gastrointestinal upset. All these might cause to miss antihypertensive medications. Additionally, when patients have additional health problem, their attention might be diverted towards the newly developed disease. However, it was inversely associated in the study in Pakistan [27]. This could be due to difference in study population and early diagnosing and treatment of co- morbidities. In the Pakistan study, participants only with essential hypertension were included. But in this study, participants with any type of hypertension were included.

Duration on anti-hypertension medications had a significant association with the outcome variables in this study. The finding is similar with those of other studies in comprehensive teaching Hospital in Shanghai, China [32], Hong Kong [25], and Singapore [28]. When patients take antihypertensive medications for a long period, they would develop awareness about the complications of missing drugs and have good attitude about hypertension treatment, and these encourage patients to adhere.

This study revealed that there was a strong association between good drug adherence and respondents’ attitude towards antihypertensive medications. This finding agreed with the finding of the study done in Hong Kong [25]. Having a positive attitude towards antihypertensive medications avoids misconceptions, which can cause non-adherence. People with a positive attitude may make accurate decisions and appropriate lifestyle modifications which may motivate adherence.

In this study, medical cost had a significant association with adherence to antihypertensive medications. This is comparable with the finding of a study done in Shanghai [32]. If the cost of the drug is affordable or free patients may take their medication regularly. This would help to have good adherence to antihypertensive medications.

This study noted the presence of significant association between patient- provider relationship and adherence status. The same finding was documented in studies conducted in Shanghai [32], and Southwest Nigeria [24]. Good patient-provider relationship would create a positive attitude towards treatment, and patients would have trust in the health care and the professionals. This would enhance adherence towards the recommended treatments.

In this study, difference in the level of drug adherence between urban and rural residents was not observed. The same finding was observed in a systematic review and meta-analysis [33]. A retrospective cohort study conducted in Canada also noted that there was no difference in drug adherence between urban and rural residents [34].

Though different studies have shown that blood pressure control status is significantly associated with drug adherence to antihypertensive medications [23, 35, 36], their association was not statistically significant in this study. This might be due to difference in the measurement of blood pressure control. In this study, the level of control was determined by using the single blood pressure measurement taken at the time of data collection; the other one might have used the average of two or more measurements taken on different appointment dates.

As a strength, the participants in this study were taken from different referral hospitals at different geographical areas. Thus, people who had different socio-demographic characteristics were involved. So, it gives evidence based information for Federal Minister of Health of Ethiopia and other stake holders and the information will be used to design strategies and take action to further improve level of drug adherence.

Since, patients attending their antihypertensive medications in health centers were not included in this study; this imposed a limitation on the generalization of the findings to all hypertensive patients. Since, the data was collected using interviewer administered technique, this study is prone to social desirability bias. However, the study participants were adequately informed about the relevance of this study and the importance of telling the truth. Additionally, the data collectors employed were working out of the chronic illness clinics.

## Conclusions

The prevalence of good drug adherence to antihypertensive medication/s was found high. Factors like co-morbidity, duration of antihypertensive treatment, medical cost, attitude towards antihypertensive medications and patient–provider relationship are important predictors of good adherence to antihypertensive medications. Prevention of co-morbidities, medical services accessibility, and having good client-provider interaction have paramount importance for good drug adherence.

## Abbreviations

AOR: Adjusted odds ratio
BP: Blood pressure
CI: Confidence interval
COR: Crude odds ratio
HTN: Hypertension
LVH: Left ventricular hypertrophy
MSc: Master of science
SD: Standard deviation
SPSS: Statistical package for social science
WHO: World Health Organization
